# Image Processing Analysis of Plant Morphometry with Examples from the Genus *Sedum* (Crassulaceae)

**DOI:** 10.3390/mps7040056

**Published:** 2024-07-24

**Authors:** Mădălina Borcă, Alexandru Borcă, Alin Ciobica, Gabriela Halitchi, Andrei Stoie

**Affiliations:** 1 Department of Biology, Faculty of Biology, Alexandru Ioan Cuza University, B dul Carol I, No. 11, 700506 Iasi, Romania; 2 Faculty of Engineering, George Emil Palade University of Medicine, Pharmacy, Science, and Technology of Targu-Mures, Gheorghe Marinescu 38, 540142 Targu Mures, Romania; 3 Center of Biomedical Research, Romanian Academy, Iasi Branch, Teodor Codrescu 2, 700481 Iasi, Romania; 4 Academy of Romanian Scientists, 54, Independence Street, Sector 5, 050094 Bucharest, Romania; 5 “Ioan Haulica” Institute, Apollonia University, Păcurari Street 11, 700511 Iasi, Romania; gabriella_halitchi@yahoo.com; 6 Faculty of Agriculture, University of Agricultural Sciences and Veterinary Medicine Cluj-Napoca, 3-5 Calea Manastur, 400372 Cluj-Napoca, Romania

**Keywords:** morphometric analysis, genus *Sedum*, image processing analyses, biological diversity, digitization

## Abstract

The complex systematics of the genus *Sedum*, the difficulties of its classification and the ambiguity of the concrete identification of the taxa brought about the need to implement a measurement system adaptable to field conditions, so as to facilitate the accuracy of data collection, avoiding the etiolation of samples and, therefore, the deterioration of the morphological structures subject to analysis. Thus, our study describes a digitization of the classic method of making measurements using millimeter paper, thus facilitating the subsequent statistical processing of quantifiable values. Depending on the number of pixels in the photos taken and the pixel/millimeter ratio, a variable measurement scale can be created depending on the size of the analyzed taxomes. The method used adds to the classic taxonomy, which is based on the analysis of morphological characteristics to determine the species of these succulent plants. The applicability of our method is shown by means of the example of an analysis performed on the flowers of the native species of the genus *Sedum* in the territory of Romania.

## 1. Introduction

The genus *Sedum* is the largest within the Crassulaceae family, encompassing 35 genera and approximately 1500 species characterized by significant morphological diversity and systematic complexity. The classification of the *Sedum* species has varied considerably over time, influenced by advances in study methods. Molecular analyses have clarified its polyphyletic nature, leading to profound taxonomic changes, approximately 420 species are currently recognized. Its extreme morphological diversity and phenotypic homoplasy contribute to the ongoing challenges for *Sedum* taxonomy [1,2]. The accurate identification and classification of the *Sedum* species are crucial for understanding their ecological roles and evolutionary relationships. Traditional morphometric methods using millimeter paper, while valuable, are often limited due to their precision and practicality in field conditions. To address these limitations, our study introduces a digitized measurement system that enhances data accuracy and facilitates the statistical analysis of its morphological characteristics. This novel approach aims to complement existing taxonomic methods and provide a more robust framework for the study of *Sedum* and other plant genera. As far as the ecology of the species is concerned, it inhabits predominately rocky areas, with a distribution in the subtropical and temperate regions of the northern hemisphere; in Europe, there are three centers of diversity (West Mediterranean, East Mediterranean and Irano-Turanian) [3]. The phenotypic diversity, correlated with the wealth of species, has caused the genus systematics to be in a constant dynamic state, and due to the aspects described above, there is ambiguity in the understanding of the genetic boundaries and cladistic relationships within the genus [4]. ‘t Hart specified in 1991 that out of the 500 species in the genus, only 54 are native to Europe, and out of these, 18 are mentioned in the Romanian flora [3]. However, one cannot exclude the possibility that, alongside these recognized taxa, there are other naturalized or resulting adventives, following the process of natural hybridization. Information on how the presence of representatives of the genus *Sedum* changes in different regions has appeared relatively frequently in the literature. The *Sedum* genus’s remarkable capacity for naturalization is recognized, and, therefore, many *Sedum* species are mentioned as alien in the flora of the following European countries: Romania [5], the Czech Republic [6], Lithuania (including *S. album*, *S. hispanicum*) [7], Bosnia and Herzegovina [8]. Similarly, the species of the genus are very easily capable of interspecific hybridization [9,10,11] and intergeneric hybridization [12], thus contributing to the modification of the flora of various regions. The issue of interspecific hybrids has not been highlighted for the taxa in the flora of Romania, although it is very likely that it exists. This aspect has been highlighted in Europe [9], for the taxa in Mediterranean areas [11,13], and also in America [14], where the authors reported ‘possible hybrids’ observed in the field during an extensive revision project of the Gormania section. Observations on the genus *Sedum* in Romania have mostly been integrated into large studies on well-defined regions [15] and have rarely constituted a point of particular interest [16,17,18]. The phenotypic variation within *Sedum’s* populations is one of the causes of the large number of infrataxon names [13,19], many of which fell into synonymy in the following decades. In the flora of Romania, this aspect has been mentioned in several articles over time [18,20]. The purpose of this study is to propose an improved measurement technique directly applicable to the field but also to contribute to the enrichment of the database regarding the chorology of the genus *Sedum* in the flora of Romania. In the field of plant systematics, morphometry can be defined as the quantitative analysis of biological forms, their morphological composition. Currently, the field of morphometry is in full development, as digitization increases its applicability and viability in terms of plant systematics. Thus, classic morphometry [21] and geometric morphometry can be distinguished [22,23]. The reference work in plant systematics using morphometry is closely correlated with the school of numerical taxonomy, whose development led to the use of morphometry and multivariate statistics [24].

### 1.1. The Importance of Morphometric Analyses in the Context of Understanding the Biology of the Genus Sedum

In this study, we made comparative analyses of the various morphological indices of flowers of the *Sedum* species from the native flora of Harghita County (Pietrele Roşii Botanical and Geological Reserve in Tulgheș, the cliffs of Toplița Municipality and the cliffs in the area of Hodoșa village), thus complementing the studies already carried out in this field of botany. The *Sedum* species present in these locations contribute, through the morphometric analyses carried out, to an understanding of the biology of the species and to the taxonomic delimitation of its possible local ecotypes. The objectives of this research include the determination of the descriptive morphological indices characteristic to the *Sedum* species and their use in the form of strings of measured values (raw data obtained from the analysis of floral morphology) in the statistical processing of data, in order to obtain information on the state of interspecific and intraspecific relationships, and in the analysis of the similarities and differences occurring between the species studied. Berger (1930) [1] relied, in principle, on three floral characteristics in order to define the subfamilies within the Crassulaceae family, namely, the haplostemonous androecia, sympetalous flowers and polymeric flowers. Hart (1984) [13] proposed that Sedum is a paraphyletic genus that is widely distributed geographically. This hypothesis was later expanded to assume that certain Sedum species appear to have given rise to other genera within the family that are limited to a specific biogeographic region. Given this, it is important to emphasize the significance of studying flora morphology in order to fully comprehend the characteristic biology of Sedum species in the autochthonous flora of Harghita County, Romania. The flower is the generative organ at the level of which the morphological characteristics of major importance for the identification of *Sedum* species are found. Knowledge of these specific morphological characteristic indexes is of great use in the delimitation and description of the possible ecotypes present in the autochthonous flora. The morphometric characteristics of the floral elements are frequently present as discriminating elements in the determination keys developed by various authors [5,14]. Along with modern analyses, classic observations made in the early stages in the field remain essential in the study of the genus Sedum (and not only), as evidenced by the large number of species new to science described in recent decades [25,26,27]. For preliminary analyses in the field, it is beneficial to select valuable material that can provide useful information or even novelties when subjected to a more thorough analysis (in the laboratory). Through the analysis of the floral characteristics of the genus *Sedum*, the verification of the morphological characteristics, well described in the determination keys of the species under study, was also performed. Thus, any considerable difference observed can then also be analyzed in the light of molecular genetics in order to establish phylogenetic links between species. Most of the time, the phenotype is described as being the result of direct interaction between the genotype and the living environment, so digitized morphometry studies should complement molecular genetics studies, resulting in a broad picture of the biology, phylogeny and ecology of the studied species. The species of the genus *Sedum* are characterized according to the literature by the following properties: 

*Sedum acre* L. is a perennial, chamaephyte, herbaceous species, found in sunny meadows, sandy places and on cliffs. It has succulent, ovate leaves with the maximum width towards the base. Yellow flowers, 6–9 mm in length. The plants reach a height of 5–15 cm and blossom in May–July. It is often confused with *Sedum sexangulare* [28].

*Sedum annuum* L., an annual or biennial species, therophyte or hemitherophyte, is common throughout Romania in mountainous regions. Has succulent, glabrous leaves; the petals are yellow, twice as long as the sepals. The plants reach a height of 6–15 cm [28].

*Sedum hispanicum* L. is an herbaceous plant, annual or biennial, therophyte or hemitherophyte, polymorphic (shows great variation in morphological characteristics). The species has succulent leaves and flowers with whitish petals, is 5–15 cm tall and blossoms in June–July. It is common throughout Romania [28].

### 1.2. Description of the Ecological Conditions Surrounding the Species of the Genus Sedum

This study was carried out in three separate locations in Harghita County, Romania, in order to highlight the morphological variations occurring between the species identified in the field. In the following, we briefly describe these areas.

1. Pietrele Roşii Botanical and Geological Reserve in Tulgheș is a protected area of national interest located in Harghita county, on the administrative territory of the Tulgheș commune. It is located at an altitude of 1215 m on the mountain Piatra Comarnicului, a small limestone massif with a maximum height of 1519 m located NE of Tulgheș and representing the SW extremity of the Bistriței Mountains. The Red Stones Nature Reserve is an area with forests and clearings, where lies a rock composed of hippuritic limestones (Cretaceous deposits of lamellibranchiata). The reserve has phytogeographical importance due to its endemic and rare species and is composed of the following phytoclimatic stages: the mountain stage of pure spruce, the mountain stage of mixed forests, the hilly stage of oaks and the hill stage of mixed forests. The local flora includes several important species of flora: *Astragalus roemeri*, *Delphinium simonkaianum*, *Hieracium pillosum*, *Silene acaulis* etc., as well as pure beech and spruce forests in the subalpine stage. The following succulent plants belonging to the *Crassulaceae* family have been identified in this reserve: *Sedum hispanicum*, *Jovibarba globifera*, *Hylotelephium* [29].

2. The cliffs of Toplița Municipality, Harghita. The Toplița depression is, in fact, the north-western part of the Giurgeului depression. It is located in the valley of the Mureş river and its tributaries, at the entrance to the Toplița-Deda gorge (40 km). It is part of the great chain of depressions that crosses the middle of the Eastern Carpathians from the Maramureş Depression to the Brasov Depression. In Toplița, the wide bed of the Mureş and the overflow points of its tributaries have formed a small depression called the Toplița Depression. The Tarnița peak has several natural groups of cliffs, which are suitable for the growth of succulent plants belonging to the *Sedum* and *Sempervivum* genera; the peak reaches an altitude of 1044 m. On these natural groups of cliffs, we find *Sedum annuum*, *Sedum hispanicum*, *Sempervivum marmoreum* and *Hylotelephium maximum* [30].

3. The cliffs in the area of Hodoșa village belong to the commune of Sărmaș in Harghita County. Near the village, there is a group of cliffs with natural-looking vegetation. On these cliffs, we find the species *Sedum acre*, which is autochthonous, and the species *Phedimus spurius*, of Caucasian origin but naturalized in Romania [31].

As a result, our study describes the use of software for morphometric analysis of the floral elements characteristic of Sedum species in order to assess potential local ecotypes. For this evaluation, three distinct locations in Harghita County, Romania (described in the previous paragraphs), were chosen, and samples were selected to highlight the morphological characteristics of the species in relation to ecological conditions. The ImageJ software can also be used in the context of analyzing other vegetative or generative organs of plants; it can also be extended to deepen studies in other biological fields such as entomology. Aside from the limitations discussed in the research materials section, this type of morphometric analysis can be easily applied in the field, using a camera rather than a binocular microscope to take photos from which measurements will be made later. Following the steps outlined in describing the proper use of the original ImageJ software (version 1.54j), measurements are made easily and accurately, and the results obtained can then be centralized in a spreadsheet for the statistics of this study. Our study aimed to emphasize the importance of digitizing existing techniques in order to facilitate the advancement of scientific fields of interest. Additional studies within the genus Sedum are required to supplement the existing information, allowing the problem of this botanical genus to be solved by combining morphometry studies with extensive molecular genetics and bioinformatics research.

## 2. Research Methods

Image processing analysis in the context of classical taxonomy involves measuring the specific characteristics of the studied taxa based on image accuracy as it can provide concrete data about the species in question.

Depending on the number of pixels in the photo and the pixel/millimeter ratio, a measurement scale can be deduced. In the case of our study, the pictures being 6000 × 4000 pixels, with a 1:1 macro lens (focus set 1:1), an accuracy of approximately 260 pixels/millimeter is obtained. 

This method can be applied both in the field on harvested material (if it is not transported to the laboratory) but also in the field directly on the analyzed taxon, without harvesting it.

Our methodology leverages high-resolution imagery and advanced image processing software (ImageJ, version 1.54j) to perform precise morphometric analysis. Photographs were taken using a DSLR camera (Nikon D3500) with a macro lens (Sigma 105 mm) at a resolution of 6000 × 4000 pixels. Calibration was achieved using graph paper, yielding an accuracy of approximately 260 pixels per millimeter. The following steps outline our approach:

Field Sample Collection:

Samples of *Sedum annuum*, *Sedum acre* and *Sedum hispanicum* were collected from three locations in Harghita County, Romania.

Image Capture:

Photographs of the floral elements (sepals, petals, stamens and carpels) were taken from frontal, dorsal and lateral views using a tripod-mounted camera set to a 90-degree angle for consistent focus.

Image Processing:

Images were processed using ImageJ software to measure 1 mm in pixels. Morphometric analysis was conducted on sepals, petals, stamens and carpels, and measurements were recorded in millimeters.

Data Analysis:

Collected data were centralized in spreadsheets and analyzed using statistical tools to identify significant morphological variations and similarities among the species.

### 2.1. Examples of Harvested Plant Samples

Photographs were taken immediately after the collection of *Sedum* species (*Crassulaceae*) samples from natural habitats, which were then used to make measurements. Morphometric analyses were carried out on the following succulent plant species: *Sedum annuum*, *Sedum acre* and *Sedum hispanicum*. Six flowers were analyzed from each collected sample and positioned in the camera field frontally, dorsally and laterally. A morphometric analysis of sepals, petals, stamens and carpels was performed using the photographs obtained. The obtained raw dataset was used for the statistical analysis of floral morphology parameters characteristic of the genus *Sedum*. The flower measurements were expressed in mm (millimeters) and were made for the following floral elements: sepal length, sepal width, petal length, petal width, carpel length, carpel width, stamen length.

### 2.2. Measuring Technique Using Pixel-Based Methods (DSLR Photographic Camera)

By using this measuring technique, we aimed to facilitate the measurement of the floral morphology of typical succulent plants belonging to the genus *Sedum* directly in the field, in the areas from which the samples were taken, as the use of a binocular microscope would limit research to the laboratory. In terms of field viability, the technique has some limitations, such as the lack of all the angles described above, because samples can only be photographed frontally in the natural setting (when referring to floral elements). As a result, in photographs where the analyzed samples are not collected or cannot be collected, calibration is performed prior to taking the photos (by selecting the Global option in the Set Scale window in the software) so that millimetric paper is not required, as the focal length is known during calibration. At the same time, technological advancements in science are critical to the progress and evolution of any field of interest. The following paragraphs describe how to use and apply the technique for determining the morphological parameters of flowers.

- Equipment used: d-SLR camera (Nikon D3500), Sigma 105 mm macro lens, tripod, graph paper;
- Biological material: flowers of *Sedum annuum*, *S. acre* and *S. hispanicum* collected from the field.

**Figure 1:**
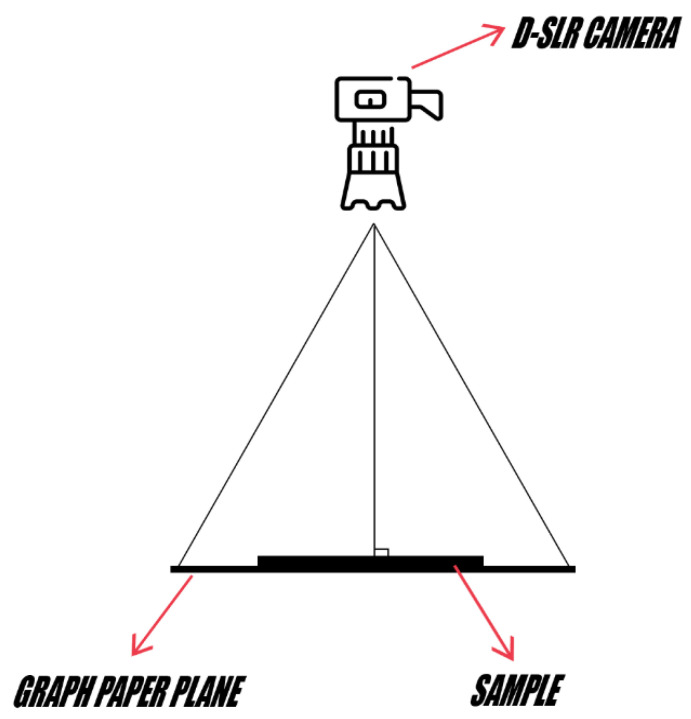
Graphic representation of the importance of the angle in the context of taking photos.

Figure 1, depiction of placing the camera at a 90-degree angle on a tripod to take photos.

### 2.3. Taking the Photographs

**Figure 2:**
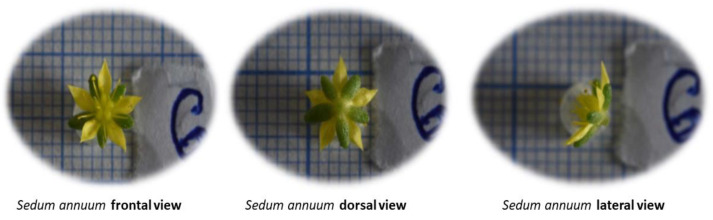
Taking the photographs.

The method of measuring sepals, petals, carpels and stamens involves mounting the camera on a tripod and setting the minimum focal length (for 1:1 macro photography). The graph paper is placed in the focus of the camera, which is positioned at a 90-degree (angle applied to the plane to be photographed, being identical to the plane of the graph paper) angle on the tripod; the flowers are then positioned frontally, dorsally and laterally, and macroscopic photographs are taken, as depicted in the following figure (Figure 2).

### 2.4. Performing the Measurement

We used the image processing software ImageJ (open source and available on all operating platforms), which can be found at the following address [32] and source code link [33]. The photos are opened and one mm (millimeters) in pixels is measured; the measurement tool is then used repeatedly in order to carry out the morphometric analysis of sepals, petals, stamens and carpels. The results are then entered into a spreadsheet.

## 3. Applicability of the Method to Species Not Harvested

**Figure 3:**
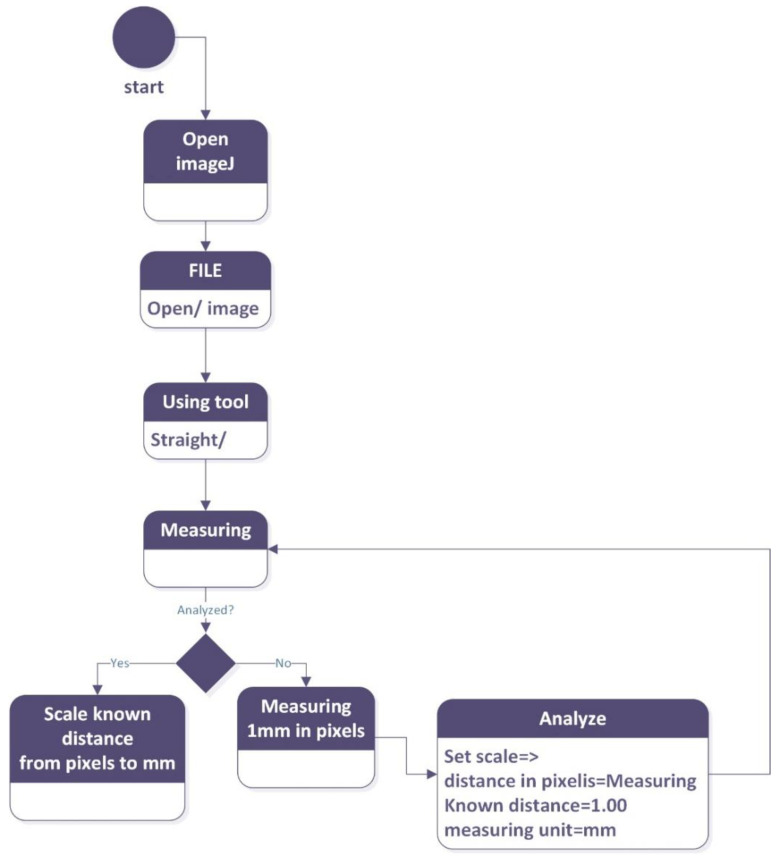
The steps that need to be executed in order to carry out measurements using ImageJ.

With the focus of the lens set, you can set the measurement scale before taking photos (Figure 3) of the species under study.

**Figure 4:**
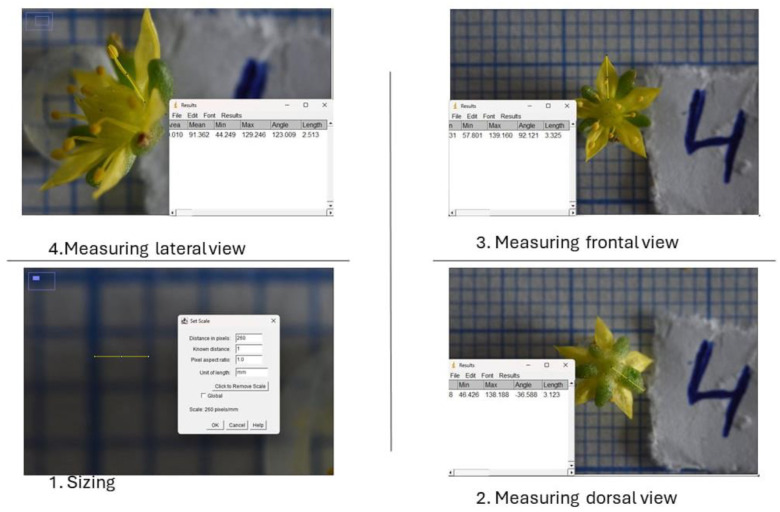
An example of measuring flower elements.

Figure 4 depicts an example of measuring flower elements.

The angle of investigation can only be frontal, given the position of the individuals in situ.

The method is harmless, in that it does not affect the biological individuals investigated.

## 4. Results and Discussion

Statistical processing was carried out using the raw data (series of measurements) that had been centralized in spreadsheets. First, analysis of variance was applied to identify whether the sample statistically significantly influences the morphological characteristics analyzed. Secondly, for comparison purposes, the mean and standard deviation of the mean were calculated. The standard deviation of the mean indicates how large the confidence interval of an empirical mean is, calculated for the measurement series, or how much it can deviate upwards or downwards (±) from the absolute value of the respective series. 

The statistics of the study were made in the program IBM SPSS [34].

We measured the variation in the floral morphological characteristics of the *Sedum* species. 

**Figure 5:**
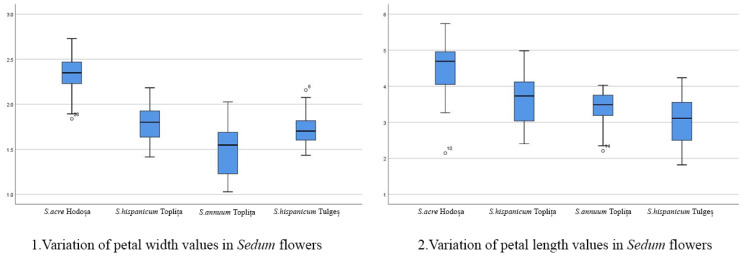
Comparative analysis of the morphology of floral elements: petals.

**Figure 6:**
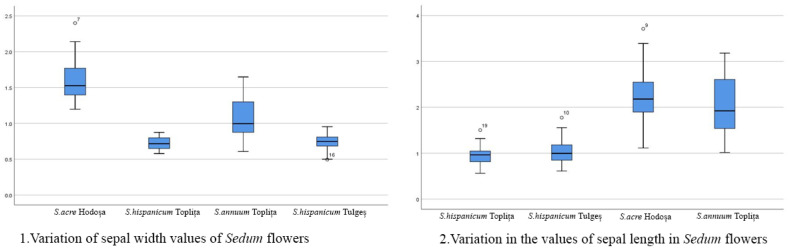
Comparative analysis of the morphology of floral elements: sepal.

**Figure 7:**
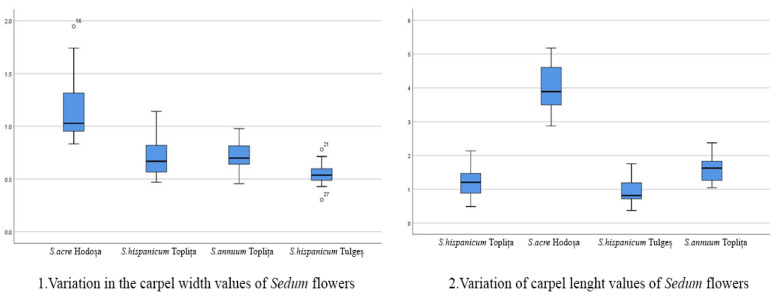
Comparative analysis of the morphology of floral elements: carpel.

**Figure 8:**
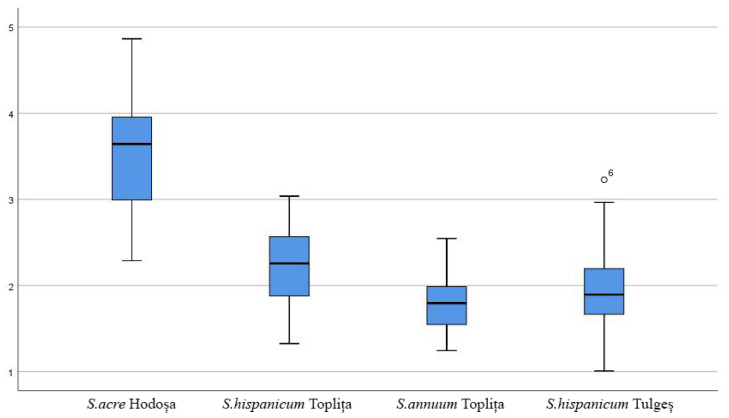
Comparative analysis of the morphology of floral elements: stamen.

The length/width ratio of sepals, petals and carpels is strictly related to the shape of these floral structures, a more elongated shape being defined by a higher ratio value. It was determined that the *Sedum annuum* from Toplița showed the highest ratio value between sepal and petal length/width, but in the case of carpels, the ratio value was higher for the *Sedum acre* from Hodoșa. The lowest ratio values were found for the *Sedum hispanicum* species. The box plot interpretation demonstrates the relatively high variability in the value series in the context of the succulent samples studied. For example, petal length and width are much greater in *Sedum acre* compared to the other species analyzed, while *Sedum hispanicum* is characterized by medium-sized petals and very small sepals. The box plots also show the maximum and minimum values, the standard deviation (interquartile range) and the mean standard deviation, as well as the points of slight abnormality. Their presence supports, both statistically and scientifically, the accuracy and viability of the measurements carried out on the floral morphology characteristics of *Sedum* species in the spontaneous flora of Harghita county. The following charts (Figure 5, Figure 6, Figure 7 and Figure 8) show the variations in the analyzed characteristics.

1. Variation in petal width values in *Sedum* flowers: Within this parameter, there are significant differences between the *Sedum hispanicum* and *S. annuum* species, but there are also notable similarities between the two distinct *S. hispanicum* samples, which is also true for the samples of *Sedum annuum* species subjected to morphometric analysis.
2. Variation in the petal length values in *Sedum* flowers: In the context of the morphometric analysis of this characteristic parameter of floral morphology, significant differences between *Sedum acre* and the other species analyzed are identified. There are also less relevant differences between the *S. annuum* species analyzed in the two particular contexts. At the same time, slightly significant differences are also present within the two distinct populations of *S. hispanicum*.

1. Variation in sepal width values in *Sedum* flowers: Similar to the previous variation in sepal length values, variation in sepal width values corresponds to the above observations: there are wide variations within the *Sedum acre* and the *S. annuum* species and less significant variations within the *S. hispanicum* species collected from the two separate locations in Harghita County. The reported variations are closely related to the structural constitution of the analyzed flowers.
2. Variation in the values of sepal length in *Sedum* flowers: Significant variations are observed in *Sedum acre* and *S. annuum*, species belonging phylogenetically to the *Acre* clade. The flowers are type 5, with yellow petals and actinomorphic symmetry, but the size of *S. acre* flowers is considerably larger compared to *S. annuum* flower sizes.

1. Variation in the carpel width values of *Sedum* flowers: In this context, significant variation was identified between the samples of *Sedum acre* and the other samples studied. Significant variations also exist within the samples of *S. annuum* species analyzed in particular contexts.
2. Variation in carpel length values of *Sedum* flowers: In the context of morphometric analysis of this characteristic parameter of floral morphology, significant differences between *Sedum acre* and the other species analyzed are identified. There are also less relevant differences between the samples of *S. annuum* species analyzed in the two particular contexts. At the same time, slightly significant differences are also present within both distinct populations of *S. hispanicum*.

Both quantitative and qualitative morphological data are essential for accurate plant species identification. Our study demonstrates that quantitative data, such as petal length and width, can effectively distinguish between species like *Sedum acre* and *Sedum annuum.* The digitized method enhances the precision of these measurements, providing robust data for statistical analysis. Furthermore, integrating these findings with molecular genetics studies could offer deeper insights into the phylogenetic relationships and evolutionary history of the Sedum species. Future research should focus on expanding this approach to other plant genera and combining it with genetic analyses for comprehensive taxonomy studies.

- Variation in stamen length values of *Sedum* flowers: This chart shows significant differences between *Sedum acre* and the other species analyzed. There are also significant variations in the two samples of *S. annuum* analyzed in separate contexts.

The morphometric analyses of *Sedum* species from the native flora showed that there were significant differences between the species investigated and less significant differences between the locations from which they were collected. For example, the length and width of petals are much greater in *Sedum acre* compared to the other species analyzed. Meanwhile, *S. hispanicum* is characterized by medium-sized petals and very small sepals. The variations identified in flower morphology are most likely due to intraspecific variability in the species analyzed and probably less to environmental conditions, and the similarities observed are probably due to these species, having well-defined botanical characteristics.

This aspect of the study emerged based on the need for a measurement technique that could be used in the field, without the need to use a binocular microscope, which can only be performed in a laboratory. For this purpose, we used a camera and a macroscopic photography lens, as well as graph paper for calibration purposes, and by using the ImageJ software, we measured 1 mm in pixels. This technique has the advantage of producing extremely small errors in the obtained values, as evidenced by their similarity to those described in the literature. This study also shows the increasing need for innovation in all scientific branches of biology, for digitization of the research carried out and, of course, for documentation that is interlinked with the field of computer science and programming.

Table 1, Table 2, Table 3 and Table 4 present the results obtained from the morphometric analyses carried out on the flowers of *Sedum* species in Harghita County, Romania. Thus, the following aspects can be observed: the variation in floral morphological characteristics depends on the species analyzed. For example, the flowers of *Sedum acre* are larger than the flowers of *Sedum annuum*, while those of *Sedum hispanicum*, which were analyzed from two distinct locations of Harghita County (Toplița and Tulgheș) to identify possible varieties or local ecotypes, also show variations in the length and width of carpels, sepals, stamens and petals, and, in terms of the number of floral elements, *Sedum hispanicum* has a varied flower morphology (with six or more floral elements). What can be seen in the adjacent tables are the raw values obtained from image processing, or the string of data that were later used in statistical processing.

## 5. Future Prospects and Possible Limitations

Regarding the applicability of the method of carrying out the morphometric analyses proposed by us, which is essentially an updated version of the measurement technique using millimeter paper, this is an optimal method for field studies, having great utility when it is necessary to study species in their own ecosystems, without allowing sampling for further studies.

An obvious advantage of the method described and exemplified above is that compared to measuring with millimeter paper alone, the accuracy of the measured values is much higher because one millimeter is divided by the number of pixels/millimeters.

The limitations arise from this point of view as photographs cannot be taken from all angles presented, but this is also a question of the specificity and objectives of the chosen morphometry study and especially of the biological material, the target species. Depending on the size of the taxon analyzed, the measurement scale varies.

The same applicability is presented in the case of species of the genus *Sedum*, succulent plants, whose samples can be easily etiolated after collection, thus decreasing the accuracy of the measured values. 

Even if the *Sedum* species analyzed in our study do not represent species of conservative interest, there is a species of *Sedum* (*Sedum brissemoretii* Raymond-Hamet) on the territory of the Portuguese archipelago Madeira that is vulnerable, according to IUCN [35,36].

Due to the complex taxonomy, there are more and more studies on the genus Sedum [37], molecular genetic studies, which present the phylogenetic links between species. We mention this aspect to emphasize the importance of botanical research of the Crassulaceae family, since classical taxonomy and molecular taxonomy complement each other, providing answers to researcher’s questions.

The technique can be applied to other areas of biology that require morphometric analysis, to determine morphological characteristics that differentiate, for example, local ecotypes of species.

The method presented by us, with exemplification on the flowers of the genus *Sedum*, aims to facilitate the use of morphometric analyses in various contexts, to complement existing studies in the fields of Botany and beyond. The digitization of the morphometric analysis method that classically uses millimeter paper can be a good opportunity to create interdisciplinary links with other topical fields such as molecular genetics or with extensive studies of plant ecology focused on the elements of biogeography. Thus, there are new perspectives that broadly describe the diversity of species in particular ecological contexts. Taking into account the aspects described above, there is an openness to digitization, but like any implemented process, this measurement method can bring a series of quantitative data that help identify the intraspecific morphological variations characteristic of the genus *Sedum*. However, this morphological characterization is not enough, and it is necessary to complete morphometry studies with those of molecular genetics, studies to confirm that the morphological variations signaled generate local ecotypes due to stationary ecological conditions. The described technique is not destructive for species whose samples are not collected from the habitat, as the analysis is carried out directly in the field. This sort of analysis can be used to generate morphometric datasets, which can then be used to examine the ranges of morphological variability in the studied species. As a result, our study on the use of measurements using digitization can serve as a starting point for future research that uses digitization to facilitate quantitative data determinations to supplement information on classical species taxonomy.

## 6. Conclusions

In conclusion, the digitized measurement system we implemented significantly enhances the accuracy and efficiency of morphometric analyses in the field. By providing precise quantitative data, this method facilitates detailed statistical analysis and supports robust species classification. Our findings underscore the potential of integrating digitized morphometry with molecular genetics to advance the taxonomy and understanding of *Sedum* species. This approach offers a promising framework for future studies in plant systematics, emphasizing the need for continued innovation and interdisciplinary research. In accordance with the biogeography, the floral traits of the species identified in the three distinct locations of Harghita County were comparatively investigated by means of morphometric analyses of sepals, petals, stamens and carpels. The comparative morphometric analyses of sepals, petals, carpels and stamens, strongly supported by the statistical interpretation of the raw data in the form of explicit graphs, demonstrate that the identified variations in floral morphology are most likely due to intraspecific variability in the analyzed species and probably less to the environmental conditions, and that the observed similarities are probably due to these species having well-defined botanical traits. At the same time, the determined floral morphological indices complement current studies on the variation in succulent plant ecotypes related to species-defining characteristics but also to elements of biogeography. However, the systematics of the genus Sedum is far from complete, which is why each study analyzing the biology, ecology, biogeography or phylogeny of species belonging to this genus of plants contributes to a better understanding of the complexity of this genus of succulents.

## Figures and Tables

**Table 1:** Variation in floral morphological characteristics in *Sedum acre* (Hodoșa) (measured in millimeters).

Sample Number/*Sedum acre* (Hodoșa)	Carpel Length	Width of the Carpel	Petal Length	Petal Width	Sepal Length	Sepal Width	Stamens Length
Flower 1	3.888	0.932	4.464	2.41	1.642	1.769	3.106
Flower 1	3.902	0.947	4.008	2.485	1.539	1.197	2.981
Flower 1	3.633	1.064	5.216	2.339	2.077	1.529	2.288
Flower 1	2.988	0.877	3.954	2.233	2.287	1.614	2.893
Flower 1	2.874	0.953	3.934	2.396	1.897	1.242	2.763
Flower 2	4.025	1.484	3.873	2.359	2.272	1.418	4.067
Flower 2	4.633	1.035	4.795	2.412	1.901	2.4	4.046
Flower 2	3.899	1.741	4.276	2.515	2.028	1.294	3.966
Flower 2	3.099	0.97	3.264	2.523	3.71	2.108	3.743
Flower 2	3.853	1.322	2.144	2.199	2.854	1.495	2.991
Flower 3	3.889	1.147	4.823	1.893	2.803	1.901	2.313
Flower 3	3.303	0.978	5.197	2.133	1.891	1.462	4.062
Flower 3	3.573	0.867	4.622	2.366	1.113	1.33	2.964
Flower 3	3.155	0.833	4.92	2.323	1.953	1.391	4.863
Flower 3	2.905	0.995	4.822	2.542	2.548	1.461	3.212
Flower 4	4.816	1.947	4.175	2.475	1.944	1.853	3.829
Flower 4	5.011	1.593	4.349	2.73	2.252	1.536	4.507
Flower 4	4.551	1.163	4.388	2.211	2.309	1.334	3.822
Flower 4	4.266	0.899	4.764	2.262	3.392	2.141	3.954
Flower 4	3.742	0.963	5.005	2.299	3.005	1.416	2.997
Flower 5	4.963	1.574	4.921	2.307	2.47	1.371	4.257
Flower 5	4.903	1.077	5.223	2.468	2.106	1.679	3.905
Flower 5	5.178	1.314	5.739	2.518	1.826	1.881	3.865
Flower 5	3.766	1.244	4.958	2.039	1.756	1.433	3.366
Flower 5	4.044	0.967	4.049	2.23	2.709	1.77	3.722
Flower 6	4.661	1.378	4.623	2.204	2.466	1.523	2.993
Flower 6	3.495	1.187	5.104	2.385	2.386	1.59	3.564
Flower 6	4.605	1.02	4.842	1.838	1.936	1.703	3.042
Flower 6	3.037	1.002	3.979	2.395	1.836	1.396	3.369
Flower 6	3.63	0.844	5.493	2.283	2.739	1.682	3.922

**Table 2:** Variation in floral morphological characteristics in the *Sedum annuum* (Toplița) (measured in millimeters).

Sample Number/*Sedum annuum* (Toplița)	Carpel Length	Width of the Carpel	Petal Length	Petal Width	Sepal Length	Sepal Width	Stamens Length
Flower 1	1.602	0.762	2.523	1.152	1.54	0.965	1.831
Flower 1	1.078	0.653	2.348	1.056	1.118	0.753	1.663
Flower 1	1.345	0.814	3.107	1.228	1.017	0.878	1.761
Flower 1	1.045	0.877	3.265	1.465	1.016	0.633	1.595
Flower 1	1.389	0.689	3.47	1.142	1.364	0.628	1.522
Flower 2	1.734	0.555	3.751	1.535	1.322	1	1.874
Flower 2	1.899	0.654	3.226	1.559	1.161	0.607	1.958
Flower 2	1.378	0.546	3.734	1.526	1.61	0.797	1.244
Flower 2	1.087	0.456	3.974	1.118	1.772	0.875	1.549
Flower 2	1.754	0.798	3.371	1.028	1.667	0.942	1.269
Flower 3	1.208	0.884	3.37	1.492	2.471	1.646	1.638
Flower 3	1.557	0.64	3.252	1.055	2.282	1.035	1.676
Flower 3	1.609	0.602	2.809	1.087	1.83	0.967	1.379
Flower 3	1.588	0.459	2.206	1.346	2.081	0.991	1.622
Flower 3	1.653	0.687	3.136	1.361	1.632	0.849	1.534
Flower 4	2.115	0.922	3.851	1.677	2.093	1.265	1.411
Flower 4	1.858	0.772	3.646	2.026	2.8	1.51	1.945
Flower 4	1.966	0.865	3.625	1.79	2.941	1.402	1.488
Flower 4	1.654	0.687	3.392	1.688	2.595	1.406	1.548
Flower 4	1.884	0.934	3.821	1.588	1.91	0.906	2.013
Flower 5	2.375	0.978	4.027	1.72	2.429	1.221	2.546
Flower 5	1.68	0.613	3.187	1.59	2.606	1.129	2.385
Flower 5	1.751	0.649	3.764	1.73	1.738	0.894	2.336
Flower 5	1.643	0.544	3.835	1.561	1.935	1.012	1.985
Flower 5	1.875	0.791	3.568	1.706	1.437	0.8	1.897
Flower 6	1.828	0.75	3.502	1.598	2.91	1.52	2.44
Flower 6	1.262	0.736	3.621	1.813	2.98	1.241	1.844
Flower 6	1.266	0.675	3.581	1.515	2.72	1.443	2.295
Flower 6	1.114	0.879	2.706	1.91	3.063	1.648	2.388
Flower 6	1.265	0.707	3.835	1.588	3.18	1.3	1.988

**Table 3:** Variation in floral morphological characteristics in the *Sedum hispanicum* (Toplița) (measured in millimeters).

Sample Number/*Sedum hispanicum* (Toplița)	Carpel Length	Width of the Carpel	Petal Length	Petal Width	Sepal Length	Sepal Width	Stamens Length
Flower 1	1.548	0.536	4.239	1.732	1.015	0.842	2.698
Flower 1	1.197	0.675	4.591	1.415	0.647	0.862	2.812
Flower 1	1.02	0.617	3.875	1.55	0.561	0.71	2.563
Flower 1	1.874	0.788	3.688	1.839	0.685	0.669	2.097
Flower 1	1.453	0.566	4.048	2.07	0.89	0.692	3.037
Flower 1	1.322	0.523	4.64	1.736	1.059	0.875	2.508
Flower 2	0.834	0.709	2.546	1.881	1.32	0.774	2.553
Flower 2	0.82	0.71	2.617	1.678	1.046	0.759	1.465
Flower 2	1.469	0.63	2.865	1.801	0.73	0.578	1.525
Flower 2	1.334	0.661	3.31	1.432	0.643	0.682	2.104
Flower 2	0.885	0.602	3.771	1.626	0.855	0.84	2.561
Flower 2	0.851	0.825	2.696	1.54	1.016	0.729	2.721
Flower 3	1.059	0.734	4.475	1.926	0.956	0.798	3.022
Flower 3	1.234	0.805	3.691	1.635	1.064	0.768	2.687
Flower 3	1.151	0.589	3.522	2.183	0.971	0.715	2.262
Flower 3	1.038	0.911	3.677	1.835	0.563	0.751	2.251
Flower 3	0.885	0.877	4.05	1.98	0.757	0.717	1.84
Flower 3	0.579	0.635	3.453	2.13	1.019	0.7	2.727
Flower 4	1.381	0.62	4.983	1.839	1.503	0.735	2.195
Flower 4	1.213	0.544	4.883	1.902	1.305	0.81	2.466
Flower 4	1.152	0.469	3.848	1.801	0.938	0.578	1.879
Flower 4	0.638	0.521	4.096	1.674	0.908	0.86	2.409
Flower 4	0.488	0.522	4.325	1.71	0.849	0.801	2.567
Flower 4	0.933	0.504	4.06	1.759	1.001	0.609	1.327
Flower 5	1.452	1.119	3.037	1.574	0.98	0.613	1.443
Flower 5	1.568	0.901	4.119	2.101	1.111	0.65	2.229
Flower 5	1.896	0.835	2.778	1.626	1.294	0.606	1.975
Flower 5	2.135	0.745	3.014	1.86	0.893	0.604	1.645
Flower 5	2.045	0.819	2.404	2.046	0.815	0.615	1.499
Flower 5	1.662	1.141	3.349	2.021	1.012	0.654	2.22
Flower 6	0.993	0.633	4.403	1.923	1.029	0.605	1.427
Flower 6	1.35	0.844	4.249	1.577	1.227	0.6	1.477
Flower 6	0.934	0.792	4.1	1.305	1.028	0.673	2.535
Flower 6	1.036	0.743	4.282	1.724	0.772	0.863	1.578
Flower 6	0.975	0.577	4.248	1.726	0.888	0.677	2.05
Flower 6	1.125	0.788	4.025	1.676	0.637	0.742	2.264
Flower 6	1.078	0.842	4.402	1.764	1.043	0.634	1.623

**Table 4:** Variation in floral morphological characteristics in the *Sedum hispanicum* (Tulgheș) (measured in millimeters).

Sample Number/*Sedum hispanicum* (Tulgheș)	Carpel Length	Width of the Carpel	Petal Length	Petal Width	Sepal Length	Sepal Width	Stamens Length
Flower 1	1.382	0.714	3.167	1.526	1.178	0.872	1.908
Flower 1	1.611	0.593	3.509	1.945	1.087	0.809	2.274
Flower 1	1.756	0.559	3.932	2.013	0.612	0.826	1.929
Flower 1	1.344	0.612	2.751	1.748	0.824	0.804	1.678
Flower 1	1.698	0.522	2.782	1.953	1	0.778	1.469
Flower 1	1.742	0.641	3.281	2.157	0.99	0.819	3.228
Flower 2	0.828	0.53	4.235	1.703	1.171	0.716	2.837
Flower 2	0.759	0.527	4.014	1.818	1.556	0.739	2.832
Flower 2	0.699	0.597	2.924	1.537	1.321	0.598	1.009
Flower 2	0.876	0.602	3.937	1.702	1.775	0.738	1.636
Flower 2	0.652	0.515	3.331	1.684	0.878	0.713	1.875
Flower 2	0.713	0.501	3.554	1.677	0.619	0.654	2.238
Flower 3	0.846	0.503	3.368	1.769	0.95	0.634	2.195
Flower 3	0.787	0.548	2.7	1.591	0.968	0.5	1.831
Flower 3	0.682	0.579	2.351	1.657	0.837	0.742	1.682
Flower 3	0.796	0.471	2.05	1.786	0.65	0.495	1.311
Flower 3	0.555	0.474	2.187	2.076	0.848	0.522	1.666
Flower 3	0.623	0.433	2.499	1.797	1.035	0.867	1.542
Flower 4	1.197	0.643	3.854	1.983	1.301	0.8	1.782
Flower 4	1.089	0.542	3.893	1.632	1.315	0.802	2.052
Flower 4	1.138	0.78	3.362	1.812	1.174	0.733	2.17
Flower 4	0.877	0.475	3.642	1.875	0.906	0.954	1.881
Flower 4	0.757	0.488	3.486	1.577	0.887	0.832	2.966
Flower 4	0.894	0.433	2.594	1.787	1.071	0.684	2.115
Flower 5	0.719	0.653	2.71	1.585	1.418	0.875	2.21
Flower 5	0.738	0.51	1.988	1.638	1.45	0.7	1.724
Flower 5	0.373	0.302	2.463	1.602	1.18	0.765	1.189
Flower 5	0.732	0.573	3.055	1.664	0.704	0.785	1.33
Flower 5	1.192	0.57	2.019	1.488	0.903	0.752	1.973
Flower 5	0.804	0.429	1.817	1.434	0.828	0.567	2.07
Flower 6	1.617	0.474	3.59	1.755	2.676	0.634	1.748
Flower 6	1.148	0.456	3.6	1.583	1.667	1.2	2.314
Flower 6	1.133	0.454	3.382	1.866	1.022	0.661	1.866
Flower 6	1.047	0.511	4.286	1.75	1.541	0.711	2.783
Flower 6	1.269	0.611	4.07	1.77	1.22	0.67	2.103
Flower 6	0.886	0.433	3.448	1.7	1.531	0.891	2.261

## Data Availability

The raw data supporting the conclusions of this article will be made available by the authors on request.
